# Interparental and Parent–Teen Relationships during Adolescence as Predictors of Intra- and Interpersonal Emotion Regulation in Young Adulthood

**DOI:** 10.3390/youth4040090

**Published:** 2024-10-03

**Authors:** Saleena V. Wilson, David E. Szwedo, Joseph P. Allen

**Affiliations:** 1 Department of Counseling, Higher Education, and Special Education, University of Maryland, College Park, MD 20742, USA;; 2 Department of Graduate Psychology, James Madison University, Harrisonburg, VA 22807, USA; 3 Department of Psychology, University of Virginia, Charlottesville, VA 22904, USA;

**Keywords:** emotion regulation, interpersonal emotion regulation, adolescence, parenting

## Abstract

Parents’ contributions to their children’s emotion regulation during adolescence has been a relatively understudied interpersonal context of development, even though parents’ roles as sources of social and emotional learning persist from childhood into adolescence and the complexity of teens’ lives grows during this time. This study aims to investigate the differential predictive utility of qualities and behaviors in interparental and parent–teen relationships during adolescence for predicting youths’ development of intra- and interpersonal emotion regulation over a 13-year period. To assess these hypotheses, data were obtained from a longitudinal, multi-method, multi-informant study of 184 adolescents (107 Caucasian, 53 African American, and 24 mixed/other ethnicity; median family income of USD 40,000–60,000/year in 1999, equivalent to about USD 75,000–112,000/year when accounting for inflation) and their parents. The results provide support for a differential pattern of prediction; qualities of interparental relationships in early adolescence were significant predictors of young adult interpersonal emotion regulation, whereas behaviors in interparental and parent–teen relationships in late adolescence were significant predictors of both young adult positive intra- and interpersonal emotion regulation. Notably, some father-reported relationship predictors during late adolescence had unexpected relations with later intrapersonal emotion regulation. The results are discussed in terms of the helpfulness of these specific relationship factors during each part of adolescence for supporting positive intra- and interpersonal emotional regulation development.

## 1. Introduction

Emotion regulation plays an essential role amid the demands of everyday life. Emotion regulation allows individuals to manage cognitive and physiological responses that can be triggered by both extraordinary and routine stressors [1,2]. Without the ability to moderate intense emotional responses, acute emotional experiences can be damaging to individuals’ physical and mental health and the quality of their relationships [3–5]. Much of the emotion regulation literature has assessed only *intrapersonal* emotion regulation, that is, how individuals regulate their own emotions without the aid of others, such as cognitive reappraisal [6–8]. Though most emotion regulation occurs in social contexts [9], when social interactions are purposefully aimed at the management of affective states, they may be described by the term *interpersonal* emotion regulation (IER) [8].

Much of the research on IER has been conducted with adults and young children. It should be noted that the age estimates that relate to distinct developmental periods often differ in various sources. Childhood may include ages ranging 3 to 11 years old in some sources, wherein ages of 3 to 8 years old would be considered early childhood and ages of 9 to 11 years old are considered late childhood, and there are slightly different age ranges in other studies [10]. The World Health Organization identifies the period of adolescence as beginning at age 10 years and ending at age 19 years [11]. This range aligns with Britannica’s definition of adulthood, which they describe as beginning at age 20 or 21 years, within which the *Journal of Adolescent Health* suggests that young adulthood exists, including ages 18 to 25 years old [12,13]. Within the adult population, some of the most profound contributions have been made with regard to coregulation and IER in romantic relationships [14,15]. However, while plenty of research has also examined emotion regulation with children and some with adolescent populations, this research often does not consider the intricacies of emotion regulation in an interpersonal context during these periods. It is also clear that, beginning in infancy, individuals learn how to regulate their emotions interpersonally through coregulation with their caregiver, defined as a shared emotional system between individuals, which allows for dyadic oscillation between affective states [16]. This early coregulation, when developed appropriately, also facilitates the creation of a secure attachment relationship between caregivers and their children [17]. An abundance of literature suggests that caregivers’ extrinsic IER toward their children in moments of intense emotion guides children’s own intra- and interpersonal emotion regulation use [18–20]. This direct engagement with children in periods of high emotion, termed emotion coaching, is the primary and most critical way that younger children learn to regulate emotions [21]. As children mature, however, they develop more complex cognitive and language skills, and these increased abilities in communication and cognition have implications for their intra- and interpersonal emotion regulation skills as they are able to understand and communicate their feelings more effectively [22,23]. Thus, these studies, and several others, indicate that the nature of emotion regulation, both in its application within the caregiver–child relationship and in general abilities, likely changes throughout development [24].

Still, the need to regulate emotional experiences is pervasive and vital across the human lifespan, and IER represents an essential component of emotional development. However, research on the interpersonal nature of emotion regulation development—in terms of both interpersonal processes and outcomes—has been infrequently extended into adolescence. The lack of research conducted during this developmental period may be a critical oversight, as both intra- and interpersonal emotion regulation skills are especially important during adolescence when youth are tasked with more complicated developmental tasks [25,26]. The current paper aims to explore this notion, focusing on the contribution of IER in caregiver relationships with offspring during adolescence to the development of their intra- and interpersonal emotion regulation in adulthood. A longitudinal, multi-method, multi-informant study of 184 adolescents and their parents, taking place in the Southeastern United States, provides data from three waves of collection across a 13-year period, representing early and late adolescence and young adulthood. Data from the first two waves of data collection will be compared for their utility in predicting intra- and interpersonal emotion regulation in adulthood.

## 2. Interpersonal Emotion Regulation in Adolescence

For 10- to 13-year-olds, the transition into adolescence, often referred to as its own period of early adolescence, involves increasingly complicated developmental tasks, including burgeoning romantic relationships, added responsibilities at home and in school, increasingly important social relationships, and newly influential social media [25,27]. Interpersonal emotion regulation skills are undoubtedly key for successfully navigating these complicated tasks and the distress that may sometimes accompany them. Furthermore, a review by Barthel and colleagues [28] suggests that use of IER facilitates greater closeness in relationships, contributes to an overall more positive affect, and further promotes the development of the intrapersonal emotion regulation skills. However, of the studies reviewed by Barthel and colleagues, few examine the effects of IER use specifically during adolescence, and none of the adolescent literature reviewed by these authors utilizes a longitudinal design [28].

Significant neurological development also promotes the use of intra- and interpersonal emotion regulation skills during adolescence. The physical and cognitive growth that individuals undergo when entering adolescence is consequential to practically all aspects of life [29]. During the transition to and throughout adolescence, the development of the prefrontal cortex enhances individuals’ capacity for higher-order processes that facilitate the regulation of emotions like cognitive control, executive functioning, and selective attention [30–32]. Silvers suggests that the salient cognitive maturation that takes place during this period also renders adolescent emotion regulation development particularly sensitive to external influences [31].

Although peers take on increased importance from middle to late adolescence, defined as ages 14 to 17 years and ages 18 to 21 years, respectively, teens across adolescence still look to their parents for emotion regulation to soothe the strong emotions associated with navigating the challenges associated with these relationships [26]. Accordingly, IER with parents at this time often occurs before or after an emotional event as opposed to the more immediate responses received in childhood [24]. Parents often engage in extrinsic IER with their teens by offering advice, which prompts adolescents to seek or avoid situations in which positive or negative emotions may be elicited (i.e., situation selection), or by soothing their teens in the aftermath of strong emotional events (i.e., response modulation). Though less common in adolescence, IER may still occur in the form of situation modification when parents or teens express their emotions to one another in the midst of an emotionally fraught situation.

However, different strategies for teaching children about emotion regulation become relevant in adolescence as compared with early childhood. Whereas emotion coaching was vital during childhood and early adolescence for children to learn to self-regulate, middle and late adolescents may also benefit from indirect teaching of specific emotion regulation strategies through modeling [33]. As teens mature into late adolescents and become more autonomous within and attuned to their social world, they are more likely to observe, understand, and learn from their parents’ relationship with one another, as well as from their specific relationship with each parent. Thus, understanding the nature of these interpersonal contexts during adolescence may be an important consideration for longitudinal emotion regulation research [26]. Not only do parent relationships provide a safe place for individuals to practice IER, but interparental relationships and interactions have been shown to have impacts on individuals’ later emotion regulation [34].

In their review, Silvers identifies a gap in the literature related to the social context of emotion regulation in adolescence, a period the author describes as socially rich [31]. Although research has extensively examined caregiver-led emotion regulation interventions with children and although there is a burgeoning body of research that has examined peer relationships in young adulthood as being valuable for emotion regulation development, the author suggests that there has been little research examining the social regulation of emotion in adolescence [31]. Silvers indicates that researchers should consider caregivers and friends as potential conduits for emotion regulation interventions in adolescence [31].

## 3. Parental Relationships as Predictors of Later Interpersonal Emotion Regulation

Morris and colleagues introduced and subsequently tested the tripartite model to describe the ways in which parents and families contribute to child and adolescent development of emotion regulation [35,36]. The authors posit three mechanisms through which children and adolescents learn about emotions: observation, emotion-related behaviors, and the family emotional climate. Specifically, children learn about emotions by observing emotion-related behaviors modeled to them by their parents (e.g., parent expressivity, emotion regulation strategies), based on the emotion-specific parenting behaviors of which they are on the receiving end (e.g., emotion coaching, reactions to children’s emotions, teaching emotion regulation strategies), and in the context of the family emotional climate, which is itself affected by parenting style, parent–child attachment, and interparental relations [35].

Importantly, while the parent–teen relationship remains incredibly influential for emotion regulation development through the family emotional climate, the parents’ relationship with one another may also prove to be important. Both represent formative experiences for adolescents, shaping what children learn from their parents and the context in which they learn it. Davies and Cummings’ emotional security hypothesis provides some evidence in support of the effect of interparental relationships on adolescent emotional development, proposing that these relationships also influence children’s sense of emotional security [37]. Specifically, the authors theorize that some forms of family conflict threaten children’s sense of emotional security, depending on the meaning of the conflict for family relations. Davies and Cummings’s emotional security hypothesis later garnered support in the literature; in multiple studies, children’s emotional security about interparental conflict has been seen to be related to their psychological [37–39]. The apparent relationship between parents’ relations with one another and children’s and teens’ emotional security, as evidenced by the emotional security hypothesis, suggests a link between parents’ social-emotional behaviors and their offsprings’ intrapersonal emotional development. Given the relational nature of these predictors, it is likely that there would exist a more direct link to behaviors in the interparental relationship and their children’s use of interpersonal emotion regulation strategies, as IER represents the fusion of social and emotional processes.

Teens in particular benefit greatly from learning how to engage in emotion regulation behaviors by observing how others engage in such behaviors. Modeling proves to be incredibly influential in teens’ development of emotion regulation strategies [36]. The context in which parents model emotion regulation strategies includes intrapersonally in distressing situations and interpersonally in interactions between parents and between parents and teens. The examples that parents provide in these circumstances may be valuable predictors of teens’ later emotion regulation skills. Though researchers have found evidence to suggest that adults’ retrospective reports regarding their childhood experience of their parents’ socialization of emotion, including discussion of and responses to their emotions, predicted their current emotion regulation skills [40], few studies have been conducted examining the longitudinal effects of parent emotion socialization during adolescence.

The tripartite model provides a structure for understanding the effects of family environment and parental relationships on the development of intra- and interpersonal emotion regulation. It describes these relationships as both a source of learning about emotions and relationships and as the context in which individuals develop the skills to regulate their emotions. Regardless of whether a factor is directly teaching individuals about emotions and relationships, however, through both observation and through the family environment, children and adolescents’ abilities to regulate their emotions are shaped by family experiences. The tripartite model represents just one of many ways in which these specific factors in parents’ relationships have been categorized in the literature. However, the current paper intends on classifying these factors, as defined by current models, more broadly in terms of relationship qualities versus behaviors. Though the study of relationship qualities and behaviors is not new, research has yet to describe parent relational factors in this manner, nor have they compared them so directly.

### Parent Relationship Qualities versus Behaviors

Attachment theorists have suggested that caregivers impact their children and teens’ emotional development through two systems: by functioning as a secure base and as a safe haven [41,42]. By serving as a secure base for children and teens, parents provide indirect guidance and encouragement to support their children’s exploration of unfamiliar environments and developmental tasks [24]. When functioning as a safe haven, on the other hand, parents provide more direct guidance for their children through extrinsic IER in times of distress [24]. These descriptions of the ways in which parenting impacts children’s emotional development provide an interesting framework for considering how relationship qualities and behaviors differentially impact emotion regulation development.

First, it is important to define exactly what is meant by qualities and behaviors. Simply put, relationship qualities are states of being and feeling in relationships, or the salient, distinctive characteristics of a parent’s relationship. Similar to the secure base described in attachment theory [41,42], relationship qualities provide a safe place for teens to seek intrinsic IER, as defined by Zaki and Williams, after engaging in activating, unfamiliar experiences [8]. Some examples of relationship qualities include warmth, parenting style, attachment style, general conflictual relations, and the emotional climate of a family. In the parent–child relationship, prominent developmental theories like attachment theory indicate that qualities of warmth contribute to positive emotional outcomes for children. In the interparental relationship, Davies and Cummings’ process model provides support for the association between marital conflict and satisfaction and emotion regulation, though this research has been conducted less frequently in adolescence [37]. Thus, positive or negative qualities in the family relationships may impact adolescents’ sense of emotional security in the family environment and, subsequently, their willingness to seek intrinsic IER from their parents [42].

Behaviors, on the other hand, are defined as what is performed in a relationship, or the specific actions one takes at times in their relationships. Likened to the safe haven described by [24], parental behaviors impact teens’ emotional development directly in the form of response modulation when teens are experiencing strong emotions and by modeling to teens how to engage in intra- and interpersonal emotion regulation, such as the strategies outlined in the extended process model of emotion regulation [43]. Examples of behaviors in parent relationships include extrinsic and intrinsic IER like emotional self-disclosure, emotional coaching, and spending quality time with loved ones. Specific behaviors modeled by parents to children are one of three ways in which Morris and colleagues discuss that children and adolescents learn about emotions [36]. In the parent–teen relationship, parents’ discussion of emotions, including emotion coaching and parents’ own negative emotional expression, has been associated with fewer internalizing symptoms and more internalizing and externalizing symptoms [44]. Regarding behaviors within families and between parents, cohesion in families during adolescence has been associated with various self-regulatory outcomes [45,46].

It is likely that relationship qualities and behaviors differentially predict emotion regulation depending on which end of adolescence which they fall (i.e., early or late), though this has yet to be explored. Parent–child relationships during early adolescence are characterized by increased conflict with and decreased reliance on parents [35]. Positive qualities in parent relationships, then, prove vital to adolescent emotional development as adolescents are less likely to rely on specific supportive behaviors, and positive relationship qualities may mitigate some of these adverse relationship behaviors during this period. Furthermore, the challenges that early adolescents encounter, including increased responsibility at school and at home, complicated social dynamics, and unfamiliar romantic relationships, can be made even more difficult without a secure base, characterized by positive qualities in parents’ relationships, to turn to in times of need [26].

In late adolescence, individuals return to expecting the same level of support they received from parents before the start of adolescence [47]. This support is likely to be more useful in the form of specific parent behaviors toward their adolescent rather than relationship qualities, as the nature of their peer relationships change during this period. Late adolescents often engage in romantic relationships characterized by increased intimacy, as opposed to the primarily affiliative romantic relationships of early adolescence [48]. Specific parent behaviors, like emotional expression, can be utilized by late adolescents in these intimate relationships to facilitate mutual satisfaction.

## 4. The Current Study

The value of understanding the ways in which parents impact the development of emotion regulation cannot be overstated. However, while intrapersonal emotion regulation has received a great deal of attention in the developmental literature, the same cannot be said for IER. Furthermore, there are generally significant gaps in the emotion regulation literature related to adolescent development of emotion regulation and the persisting impact of parents in this period. The current paper aims to extend the study of emotion regulation by considering both early and late adolescent predictors of young adult emotion regulation. The nature of IER in parental and parent–teen dyads in adolescence and the relative temporal relevance of different predictors of intra- and interpersonal emotion regulation across adolescence will be explored. More specifically, the current paper examines the interpersonal role that parents play in the development of youths’ intra- and interpersonal emotion regulation and the differential predictive utility of emotional relationship qualities and behaviors in interparental and parent–teen relationships across adolescence.

**Figure 1.**
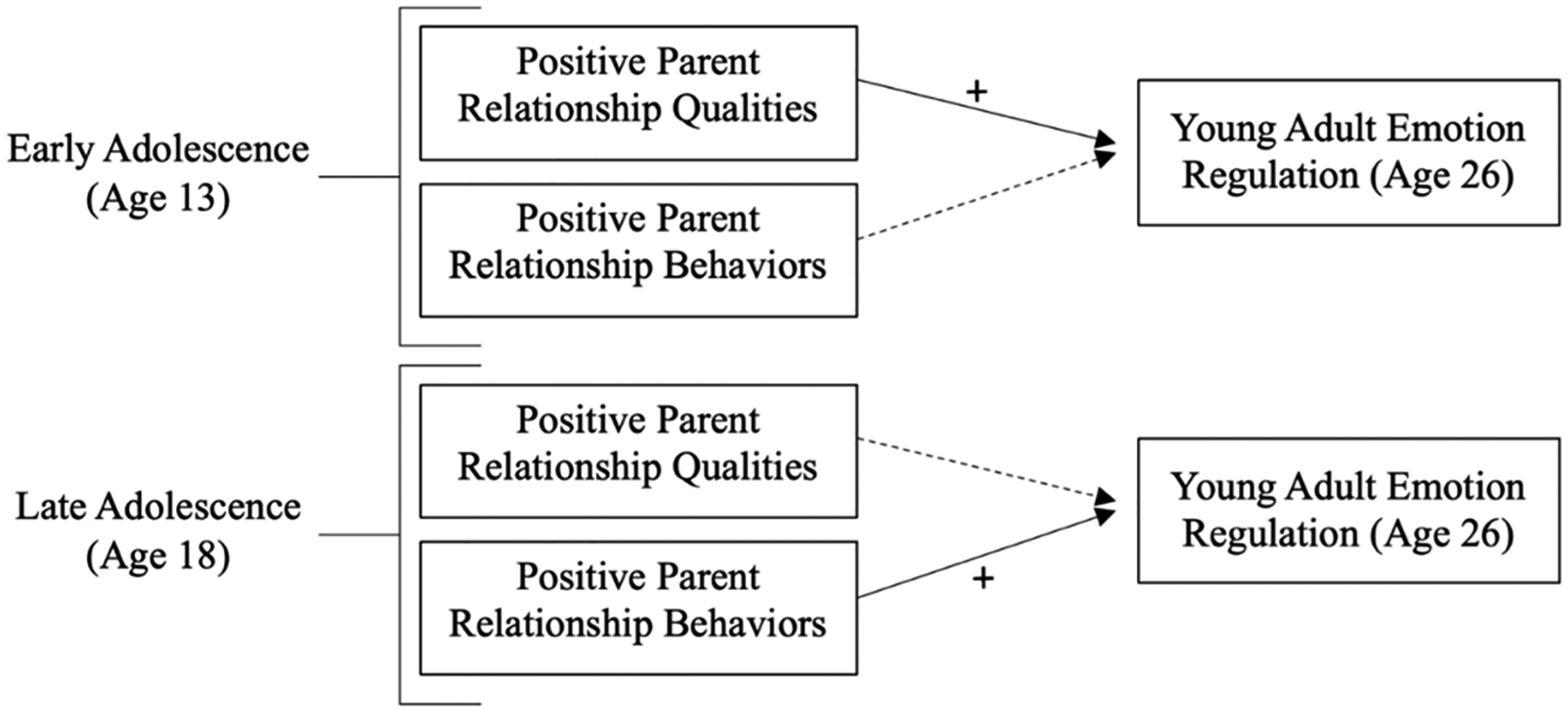
Heuristic representation of Hypotheses 1 and 2.

Given the developmental tasks of early adolescence, interparental and parent-teen relationships with positive emotional qualities are hypothesized to be stronger predictors of intra- and interpersonal emotion regulation as they provide evidence of a safe emotional space from which teens may begin to explore new emotions and relationship experiences. In late adolescence, on the other hand, positive interparental and parent–teen emotional behaviors in their relationships are hypothesized to be stronger predictors as teens become involved in more complex relationships and challenges and may benefit from parents’ modeling of emotion regulation behaviors in relationships. More specifically, the following is hypothesized (see Figure 1 for reference): (1) positive qualities of parental relationships will predict more positive intra- and interpersonal emotion regulation in young adulthood when they are reported in early adolescence; and (2) positive parent behaviors in relationships will predict more positive intra- and interpersonal emotion regulation in young adulthood when they are reported in late adolescence.

## 5. Method

### 5.1. Participants and Procedure

Participants in this study are drawn from a larger, ongoing longitudinal study of adolescent and young adult social and emotional development. Data collection for the larger study began in 1999, when participants were approximately 13 years old, and has continued on a yearly basis. The sample is composed of 184 adolescents, 85 males, and 99 females. The sample is diverse and representative of the area from which it was collected with respect to participant race/ethnicity (107 Caucasian, 53 African American, 2 Hispanic/Latino, 2 Asian American, 1 Native American, 15 mixed ethnicity, and 4 “other”) and socioeconomic status (median family income of USD 40,000–60,000/yr. in 1999, equivalent to about USD 75,000–112,000/year when accounting for inflation) [49].

Participants were recruited from a local middle school in the Southeastern United States that draws from suburban and urban populations. All parents of students in the seventh and eighth grades at the school were sent an initial mailing, giving them the opportunity to opt out of further contact (N = 298), and 2% of parents opted out at this time. Families who indicated interest were then contacted by phone and, of those eligible, 63% agreed to participate as either a target participant or as a peer providing additional information about the target participant. The sample was comparable with the overall population of the school regarding racial/ethnic makeup and socio-economic status. When participants were under age 18, participants provided informed assent before each interview session, and parents provided active, informed consent. At age 18 and beyond, participants provided informed consent. Parents, target adolescents, and peers were all paid for their participation. Transportation and childcare were provided if necessary. This study was reviewed and approved by the university’s Institutional Review Board.

For the current study, data from three waves of study were utilized. Variables assessing parental relationship qualities and behaviors were collected in early adolescence, when participants were approximately age 13 in 1999, and in late adolescence, when participants were approximately age 18 in 2004. These measures, to be described further below, asked parents to complete self-report measures regarding their marital relationship (i.e., consensus and cohesion) and to participate in an interaction task with their adolescents, assessing parent–teen self-disclosure and valuing. When participants were age 26, they completed self-report measures regarding their use of intra- and interpersonal emotion regulation strategies during times of stress (i.e., acceptance, denial, instrumental support seeking, and emotional support seeking).

### 5.2. Measures

#### 5.2.1. Interparental Relationship Qualities and Behaviors

The Dyadic Adjustment Scale (DAS) is a 32-item measure completed by teens’ parents when teens were age 13 and 18 regarding their martial quality with their current partner [50]. The consensus and cohesion subscales of the measure were utilized. The consensus subscale asks parents to rate how often they and their partner disagree about 15 different topics (finances, household tasks, goals, in-laws, etc.) on a 6-point Likert scale ranging from 1, always agree, to 6, always disagree. The consensus scale was used as a marker of relationship quality, acting as a context of development that may allow or disallow for teens to seek IER in relationships. The cohesion subscale was also utilized, identified as parent behavior. The cohesion subscale first asks parents if they engage in outside interests together on a 5-point Likert scale ranging from 0, none of them, to 4, all of them. Additionally, the subscale utilizes 4 additional items that ask participants how often different events occur between them and their partner (laughing together, exchanging ideas, working together, calmly discussing) on a 6-point Likert scale ranging from 1, “Never”, to 6, “More Often”. The cohesion subscale measures the extent to which parents engage in various behaviors and activities with one another, and these behaviors are theorized to act as a model for IER at the situation selection and modification and response modulation levels of Gross’ process model [43]. The DAS has shown great overall reliability, with Spanier reporting a total internal consistency of *α* = 0.96 [50]. In the present study, at both ages 13 and 18, respectively, maternal consensus (*α* = 0.91, *α* = 0.92) and cohesion (*α* = 0.83; *α* = 0.84) demonstrated good reliability, as did paternal consensus (*α* = 0.88; *α* = 0.94) and cohesion (*α* = 0.73; *α* = 0.83).

#### 5.2.2. Parent–Teen Relationship Qualities and Behaviors

The Supportive Behavior Task (SBT) is an 8 min interaction task with teens and parents in which teens present a problem to parents that they want advice or support about [51]. Common topics that teens brought to parents included dating, peer or sibling relationships, money, and sports teams. Codes of parents’ valuing and self-disclosure when teens were aged 13 and 18 were utilized. The valuing subscale, identified as a parent relationship quality, assessed the extent to which parents demonstrated that they care about, value, or genuinely like their adolescent. Coding of parents’ valuing ranged from 0, unclear whether the person likes the other, to 4, the parent’s behavior is overall quite warm and fuzzy, the affection and liking is strong and clear, and the adolescent knows their parent really cares about them. The self-disclosure subscale, identified as a specific parent behavior, assessed parents’ sharing of information about themselves that allowed the adolescent to know them better. Coding of parents’ self-disclosure ranged from 0, sharing briefly about likes and dislikes or one’s day, to 4, sharing about areas not commonly shared between somewhat close friends, expressing strong feelings, or sharing unusual or embarrassing information about the self. Parents’ responses to teens’ problems in the interaction task may or may not constitute IER; however, the specific subscales utilized in this study are intended to assess parent’s engagement in IER with their teens in the form of qualities (situation level IER) and behaviors (response level IER) [43]. Importantly, codes for fathers’ self-disclosure and valuing were not available when these variables were first assessed, when teens were aged 13. Therefore, father self-disclosure and valuing were not included in the analyses during this wave of data collection. Reliability was computed using intraclass correlation coefficients: (mother valuing age 13 *ICC* = 0.78, age 18 *ICC* = 0.72; mother self-disclosure age 13 *ICC* = 0.77; age 18 *ICC* = 0.84; father valuing age 18 *ICC* = 0.69; father self-disclosure age 18 *ICC* = 0.60).

#### 5.2.3. Intra- and Interpersonal Emotion Regulation

The COPE inventory is a 60-item measure developed by Carver and colleagues to assess adaptive and dysfunctional responses to stress [52]. For the purposes of this study, coping is used as a proxy of emotion regulation. While studies suggest that these constructs are distinct, they share many similarities. A key distinction between the constructs lies in the types of emotional events that trigger emotional responses; while emotion regulation is an ongoing process in which emotions are regulated under stressful and non-stressful conditions, coping represents emotion regulation that occurs under stress [53]. Still, both constructs represent regulatory processes, include purposeful efforts, and unfold and change over time. The use of coping for a proxy of emotion regulation in this particular study is supported by the developmental period that is being assessed by the measure. While a great deal of research on emotion regulation has focused on infancy and early childhood, the construct of coping is typically studied in later childhood, adolescence, and adulthood.

At ages 21 and 26, participants completed the self-report measure by rating what they usually do in response to experiencing stressful events on a 4-point Likert scale ranging from 1, “I usually don’t do this at all”, to 4, “I usually do this a lot”. In the current study, the acceptance and denial subscales of the measure were utilized to represent intrapersonal emotion regulation strategies and use of emotional social support, and the use of instrumental social support subscales were utilized to represent IER strategies. Carver and colleagues report good reliability for the COPE inventory subscales utilized in the current study (acceptance: *α* = 0.65; denial: *α* = 0.71; emotional support: *α* = 0.85; instrumental support: *α* = 0.75) [52]. In the present study, all scales of interest demonstrated adequate reliability at both ages 21 and 26, respectively: acceptance (*α* = 0.68, *α* = 0.74), denial (*α* = 0.77; *α* = 0.73), emotional support (*α* = 0.86; *α* = 0.88), and instrumental support (*α* = 0.81; *α* = 0.88).

### 5.3. Data Analysis and Interpretation

All variables in the current study were first examined to ensure adherence to the assumptions of maximum likelihood estimation and to assess bivariate relationships among variables of interest (see Tables 1–3). Full information maximum likelihood (FIML) methods were utilized for all study analyses. FIML accounts for biases in estimation that may result from missing data and allows for the least biased estimates by using all available data for longitudinal analyses [54]. Attrition analyses indicated no differences in any study variables for participants who did vs. did not have data at age 26. As a result, data were utilized for all 184 study participants; there were no data manipulations or exclusions.

A series of hierarchical multiple regression analyses predicting acceptance, denial, instrumental support seeking, and emotional support seeking were conducted in three steps. At step one, control variables (sex, income, and outcomes measured at age 21) were entered into the equations to limit the influence of potentially conflating influences on study outcomes. At step two, predictors contrary to study hypotheses (i.e., behaviors first in early adolescent models, qualities first in late adolescent models) were entered into the models. At step three, predictors in accordance with study analyses were entered into the models. Tables 4–9 include the regression coefficients of variables at both entry to the model and in the final model that consider all variables together. Beta entry values represent the regression coefficients of variables most recently entered in a model and those entered before. This allows us to examine the effects of parent relationship qualities and behaviors on intra- and interpersonal emotion regulation separately in each model. Furthermore, estimation of values in full models show the effects of hypothesized-stronger predictors beyond what is accounted for by hypothesized weaker predictors. Early and late adolescent predictors were entered into separate models predicting each emotion regulation strategy separately

### 5.4. Transparency and Openness

We report within this manuscript how we determined our sample size, all data exclusions, all manipulations, and all measures in the study, and we follow JARS [55]. The raw data, analysis code, and research materials in this study are available by request to the corresponding author. Data analysis was conducted via computer software (SAS 9.4). This study’s design and its analysis were not pre-registered.

## 6. Results

### 6.1. Preliminary Analyses

#### Univariate and Correlational Analyses

Tables 1–3 display the descriptive statistics (i.e., means, standard deviations, minimums, and maximums) and correlations for all study predictors. Preliminary analyses found significant, positive relationships between income and predictor variables of parent reports of their consensus in their relationship when teens were age 13, mother valuing in interactions with teens aged 13, and father relationship variables with teens and relationship partners at age 18. Examining outcome variables, significant positive relationships were found between sex and the use of IER strategies such that being female was associated with a greater use of IER strategies. Significant positive relationships were also found between income and the use of intra- and interpersonal emotion regulation strategies. These results are presented in Table 3. Because of the significant relationships found in preliminary analyses, sex and income were controlled for in all study analyses. Regression analyses also included variables assessing each corresponding outcome assessed at its earliest time available in the study (age 21) to act as a baseline measurement of intra- and interpersonal emotion regulation. This allowed us to examine whether relationship qualities and behaviors could predict later intra- and interpersonal emotion regulation independently of participants’ pre-existing emotion regulation at age 21.

### 6.2. Primary Analyses

#### Multivariate Regression Analyses

##### Hypothesis 1. Positive qualities of parental relationships will predict more intra- and interpersonal emotion regulation in young adulthood when they are reported in early adolescence.

In early adolescent models, positive interparental relationship *qualities* predicted a relative increase in positive intra- and interpersonal emotion regulation strategies during young adulthood. Greater father-reported interparental consensus predicted youths’ increased use of emotional social support (*β* = 0.21, *p* = 0.044; see Table 4), and greater mother-reported interparental consensus predicted increased use of acceptance of emotions (*β* = 0.27, *p* = 0.034; see Table 5). There were no significant parent relationship predictors at age 13 for the use of denial or instrumental support as emotion regulation strategies at age 26.

##### Hypothesis 2. Positive parent behaviors in relationships will predict more intra- and interpersonal emotion regulation in young adulthood when they are reported in late adolescence.

In late adolescent models, positive interparental and parent–teen relationship *behaviors* were primarily strong predictors of adaptive intra- and interpersonal emotion regulation. Greater father-reported cohesion between parents predicted increased the use of both emotional and instrumental support during young adulthood (emotional: *β* = 0.30, *p* =.031; instrumental: *β* = 0.33, *p* = 0.035; see Tables 6 and 7, respectively). Greater mother-to-teen self-disclosure predicted youths’ increased the use of acceptance and decreased the use of denial of emotions (acceptance: *β* = 0.30, *p* = 0.019; denial: *β* = −0.30, *p* = 0.016; see Tables 8 and 9, respectively). However, some qualities and behaviors from fathers in late adolescence predicted the increased use of maladaptive intrapersonal emotion regulation strategies; as can be seen in Table 9, greater engagement in denial was predicted by greater father self-disclosure to teens (*β* = 0.29, *p* = 0.03) and greater father-reported consensus between parents (*β* = 0.36, *p* = 0.003).

## 7. Discussion

The present study sought to compare the predictive utility of positive interparental and parent–teen relationship qualities and behaviors for predicting intra- and interpersonal emotion regulation tendencies in young adulthood. Study hypotheses were grounded in previous research and theory suggesting that adolescents’ social and emotional development are largely impacted by familial relationships and that there are differing social and emotional tasks on opposing ends of adolescence [35,37,41,42,48]. Results generally provide support for the study hypotheses. Parental relationship *qualities* were stronger predictors of intra- and interpersonal emotion regulation in early adolescence, and relationship *behaviors* appeared to come online as predictors in late adolescence.

Attachment theory suggests that individuals’ emotional security and regulation is built through early parent–child interactions that form internal working models from which individuals continue to operate and apply to new situations throughout their lives [41,42]. An individual’s attachment relationship to their caretaker depends on their ability to rely on their caretaker as both a secure base and as a safe haven [41,42,56]. Functioning as a secure base for one’s children requires providing a safe place for children to return to upon exploring unfamiliar experiences and environments [24]. The largely unfamiliar developmental tasks of early adolescence would, then, seem to necessitate a caretaker relationship, which includes several positive relationship qualities that enable the relationship to act as a secure base [26,48]. Attachment relationships that function as a safe haven require caretakers to provide direct support to children in times of distress [24]. The increasingly complicated and potentially distressing social and romantic contexts in which late adolescents are involved would, then, seem to necessitate a caretaker to engage in the direct modeling of positive emotion-related behaviors to enable the relationship to act as a safe haven for late adolescents [26,48]. Indeed, the current study found that, in early adolescence, parental relationship *qualities* were significant predictors of young adult emotion regulation beyond what was accounted for by parent relationship behaviors, and in late adolescence, parental relationship *behaviors* were significant predictors of young adult emotion regulation beyond what was accounted for by parent relationship qualities. These findings further previous research examining the impact of parental relationships and family factors on children and adolescents’ development of intra- and interpersonal emotion regulation by revealing a differential pattern of effects for parental relationship qualities and behaviors. Though previous literature has established the impact that parent relationships have on emotional development, this research has less frequently been conducted in adolescence. Furthermore, disparate ends of adolescence have yet to be explored regarding the unique emotional needs of early and late teens.

The parental relationship quality of general agreement between parents in early adolescence, interparental relationship consensus, was the only significant predictor of young adult emotion regulation in early adolescent models. Observations of mothers’ valuing of teens in their interactions were not significant predictors of intra- or interpersonal emotion regulation. Thus, when early adolescents observe positive qualities in their parents’ relationship, as opposed to when they experience positive qualities within the parent–teen relationship, they are more likely to engage in positive emotion regulation strategies. In late adolescent models, *behavioral* engagement between parents, interparental cohesion, was a significant predictor of interpersonal emotion regulation in young adulthood. These results provide further evidence for the well-documented impact that interparental relationships have on the adjustment of children and adolescents, as evidenced in the emotional security hypothesis [37]. Researchers have suggested that the effects of interparental conflict on emotion regulation may also operate through parent–child relationships factors, like attachment and parent warmth [57,58]. Other researchers have suggested that children’s responses to interparental conflict are dependent upon their emotion regulation [59]. The results of the current study extend such findings and suggest that positive aspects of interparental relationships, rather than just interparental conflict, may also play a role in social and emotional development.

Continuing with late adolescent predictors, the current study also found that mothers’ self-disclosure to teens predicted adaptive intrapersonal emotion regulation through a greater acceptance of and less denial of emotions in young adulthood. However, fathers’ self-disclosure to teens predicted maladaptive intrapersonal emotion regulation use through a greater denial of emotions in young adulthood. Disparate findings regarding the effects of parent self-disclosure may relate to the existing relationship between teens and parents at the time of or to topics which were disclosed during the Supportive Behavior Task [51]. Though there has been little research conducted on the effect or process of parents’ self-disclosure to children and adolescents, and that which has been conducted may be outdated, general self-disclosure research can be utilized to understand the results of the current study. The literature suggests that perceptions of appropriateness and responses to self-disclosure by those on the receiving end depends upon the closeness of the relationship between these two people, such that receivers of self-disclosure view self-disclosure as warm and appropriate in the context of close relationships but maladjusted and inappropriate outside of such close relationships [60]. Thus, it may be that fathers engaging in more revealing self-disclosure with teens may not have close enough relationships for such self-disclosure to be viewed positively, whereas mothers may have closer relationships with their teens in early adolescence, allowing self-disclosure to strengthen feelings of warmth and closeness within the dyad and promote greater regulation in young adulthood. This aligns with findings that suggest that mothers are perceived by late adolescents as more supportive than fathers [44] and that mothers engage more and are more involved with their adolescents (i.e., spend more time with, talk more with) than fathers [61,62].

Importantly, the current study did not examine the topics of parents’ self-disclosure during the interaction task but rather the level of self-disclosure, with more private or unusual topics shared being rated higher on this scale. A study conducted by Stern and colleagues may provide insight regarding why the topic of parents’ self-disclosure may be notable [63]. This study examined the efficacy of an intervention intended to repair attachment relationships between parents and adolescents. The study found that, in successful sessions, self-disclosure was viewed by raters as warm, disclosing, and expressing; in the unsuccessful session, parents’ self-disclosure was rated as hostile, sulking, and scurrying [63]. Hence, it may also be that the topic of fathers’ self-disclosure was similar to that examined by Stern and colleagues, hostile, sulking, and scurrying, resulting in the destabilization of or representing already unstable relationships between teens and fathers [63]. As a result, self-disclosure would not prove to be the positive relationship behavior and learning opportunity that it was proposed to be and would not be expected to be predictive of adaptive functioning in adulthood. Should future researchers wish to examine associations between parent’s self-disclosure and emotion regulation, these researchers should consider both the content of self-disclosure and the existing relationship between parents and teens.

Notably, the results from the current study have practical implications regarding the respective focus of the parenting environments in early and late adolescence. Parents who create a secure base for their early adolescents to return to, composed of positive qualities, may support more positive emotion regulation development. In late adolescence, parents who model positive relationship behaviors and provide direct support through the creation of a safe haven may promote more positive emotion regulation development. Therefore, parents should consider the overall qualities of their relationships with one another when teens are younger, ensuring to maintain consensus so as to facilitate emotional security in early adolescents. Parents of older adolescents should consider making explicit efforts to provide support for teens and model to them appropriate relationship behaviors. Positive interparental relationships composed of both general agreement and behavioral engagement may have specific positive implications for teens’ later use of adaptive emotion regulation strategies in young adulthood. Parents should consider how the positive and negative aspects of the interparental relationship impact teens’ overall emotional development. Lastly, parents who engage in self-disclosure with their adolescents in response to adolescents’ seeking of advice or support should consider both the strength of their existing relationship with adolescents as well as the topic of self-disclosure.

The implications of this study also extend to the area of clinical practice. The current study provides evidence that parents may be valuable conduits for delivering emotion regulation interventions in adolescence. Furthermore, the results of the current study cement the value of working with both parents and teens throughout adolescence when working to foster emotional regulation strategies in individual clinical work with youth. Clinicians developing emotion regulation-related interventions for parents to deliver to teens, and those working directly with teens and families, may benefit from considering the qualities and behaviors parents engage in at different developmental stages, as well as the overall impact of the interparental relationship. Efforts made to foster greater emotion regulation capacity, then, may consider the role that interparental relationships play and encourage parents to maintain positive qualities like consensus when clients are early adolescents and enact positive behavioral engagement with one another when clients are late adolescents. Within the parent–teen relationship, interventions that utilize self-disclosure to facilitate closeness and regulation capacities should consider the content of self-disclosure as well as the existing relationship between parents and teens.

There were several strengths to the study that should be noted. Most importantly, the multi-method and longitudinal design represented significant strengths. The use of interaction tasks to assess parent–child relationships allowed for less biased, applied measurements of parents’ interactions with teens. The use of multiple reporters in assessing interparental relationship factors provided the opportunity to examine how each parents’ report of their dyadic relationship provides different information about the impact of interparental relationships on teens’ social and emotional development. As it relates to data analysis, potential correlates (i.e., sex and income) were controlled for in all study analyses to account for any possible effects that these demographic factors might have on the use of emotion regulation strategies. Furthermore, all study analyses included measures of each outcome 5 years prior to the period examined in the current study to allow the authors to examine the impact of parental relationships on emotion regulation above and beyond stable emotion regulation tendencies.

There are also several limitations in the current study that are of note. Importantly, causal claims cannot be drawn from naturalistic, longitudinal study designs due to the correlational nature of the data. Furthermore, some study variables rely solely upon self-report data, which may bias or contribute to general inaccuracies in the data. Additionally, recent directions in relational literature have emphasized the bidirectional nature of parent–child relationships [26]. This research suggests that disagreement in reports within dyads may mean that neither report is an accurate depiction of the construct, suggesting that parents’ reports of their dyadic functioning in and of themselves may not provide a complete understanding of relationship qualities and behaviors within the dyad. Regarding the inclusion of earlier measurements of outcome variables, though this represents a strength to the analyses conducted, such controls would have benefitted from even earlier measurement to ensure that the comparison of emotion regulation across both time periods accurately accounted for stable emotion regulation across development. Notably, father interaction variables were not available during the early adolescent wave of measurement. This precluded the authors from examining and comparing father–teen qualities and behaviors in early adolescence as predictors of young adult emotion regulation. Results showed insignificant findings for the prediction of emotion regulation by mother valuing in interactions with teens. Thus, researchers may consider examining other parent–teen relationship qualities in early adolescence or father valuing in early adolescence, as it is possible that insignificant results are specific to valuing and/or the mother–teen relationship. Furthermore, the father report and father interaction variables often had lower sample sizes than other variables utilized in the study, making results related to fathers somewhat more challenging to interpret and generalize.

Future research may take several directions based on the results of the current study. The results of the current study suggest that developmental tasks at opposing ends of adolescence may pose different demands from parental relationships. While compelling, further research is needed to confirm this pattern. This research may consider examining such patterns with other social and emotional developmental outcomes, such as social competence and emotional awareness. Researchers may also consider how other parental relationship qualities and behaviors that have established effects on emotion regulation, like parental conflict and discussion of emotion, fall into the pattern established by the current study. More research on the impact of positive interparental relationships, as opposed to negative ones, is further implicated by the present findings. Researchers may consider how other positive relationship qualities and behaviors between parents, like intimacy and affection, impact the social and emotional development of adolescents.

## Figures and Tables

**Table 1.** Descriptive statistics for study variables.

Variable	*N*	Mean	*SD*	*Min*.	*Max*.
Sex (85 male, 99 female)	184	-	-	-	-
Income	184	USD 40–60 k/yr.	-	<USD 5 k/yr.	<USD 60 k/yr.
*Age 13 Predictors*					
Mother-reported consensus	184	41.37	13.16	24	65
Father-reported consensus	184	38.68	11.90	27	65
Mother-reported cohesion	128	15.24	4.22	4	24
Father-reported cohesion	99	14.64	3.46	6	23
Mother valuing	168	2.00	0.99	0	4
Mother self-disclosure	168	0.50	0.75	0	4
*Age 18 Predictors*					
Mother-reported consensus	108	44.90	11.97	14	65
Father-reported consensus	85	48.77	8.53	13	65
Mother-reported cohesion	109	16.17	4.76	4	26
Father-reported cohesion	82	14.90	3.96	6	24
Mother valuing	103	2.10	0.80	0	4
Father valuing	66	1.95	0.72	0.5	4
Mother self-disclosure	103	0.27	0.51	0	2
Father self-disclosure	66	0.23	0.46	0	1.75
*Age 21 Outcomes*					
Acceptance	163	6.30	2.61	0	12
Denial	165	2.00	2.37	0	10
Emotional support seeking	165	7.35	2.99	0	12
Instrumental support seeking	165	7.50	2.82	0	12
*Age 26 Outcomes*					
Acceptance	144	6.90	2.73	1	12
Denial	144	1.31	1.89	0	8
Emotional support seeking	144	7.25	3.05	0	12
Instrumental support seeking	146	7.72	2.83	1	12

**Table 2.** Correlations Between Study Predictors and Age 26 Outcomes.

		Interpersonal Emotion Regulation		Intrapersonal Emotion Regulation	
Variable Type	Variable	Instrumental Support Seeking	Emotional Support Seeking	Denial	Acceptance
Controls	Sex	0.20 *	0.32 ***	0.04	0.02
	Income	0.21 *	0.21 *	−0.02	0.20 *
Age 13 Predictors	*Relationship Qualities*				
	Father consensus	0.29 ***	0.30 ***	−0.09	0.12
	Mother consensus	0.16	0.18 *	−0.06	0.27 **
	Mother valuing	0.14	0.08	0.08	0.03
	*Relationship Behaviors*				
	Father cohesion	0.17	0.15	−0.01	0.09
	Mother cohesion	0.24 *	0.21 *	−0.01	−0.02
	Mother self-disclosure	0.02	0.02	−0.12	0.11
Age 18 Predictors	*Relationship Qualities*				
	Father consensus	0.08	0.10	0.12	0.04
	Mother consensus	0.02	0.02	0.03	0.05
	Father valuing	0.07	0.02	0.30 *	0.06
	Mother valuing	0.09	0.07	−0.17	0.05
	*Relationship Behaviors*				
	Father cohesion	0.24 *	0.23	−0.09	0.03
	Mother cohesion	0.17	0.18	0.06	−0.04
	Father self-disclosure	−0.08	0.03	0.28*	0.08
	Mother self-disclosure	−0.11	−0.11	−0.19	0.21

Note.

* *p* < 0.05,

** *p* < 0.01,

*** *p* < 0.001.

**Table 3.** Correlations among study predictors.

	1	2	3	4	5	6	7	8	9	10	11	12	13	14	15
1. Sex	-														
2. Income	−0.12	-													
Age 13 Predictors															
3. Father consensus	−0.07	0.43 ***	-												
4. Father cohesion	0.01	0.05	0.36 ***	-											
5. Mother consensus	0.12	0.32 ***	0.46 ***	0.15	-										
6. Mother cohesion	−0.00	0.02	0.18 *	0.44 ***	0.50 ***	-									
7. Mother valuing	−0.01	0.20 *	0.23 **	−0.03	0.21 **	0.17	-								
8. Mother self-disclosure	0.09	0.08	0.06	0.15	−0.03	−0.03	0.27 **	-							
Age 18 Predictors															
9. Mother valuing	0.04	0.09	0.22 *	0.20	0.02	0.28 **	0.23 *	0.28 **	-						
10. Mother self-disclosure	−0.18	−0.11	−0.00	0.19	0.06	0.07	0.05	0.13	0.41 ***	-					
11. Father self-disclosure	−0.03	−0.03	0.14	0.08	−0.02	0.11	−10	−0.00	−0.02	0.19	-				
12. Father valuing	0.01	0.25 *	0.22	0.14	0.11	0.29 *	0.15	0.06	0.25	0.21	0.32 **	-			
13. Mother consensus	−0.01	−0.13	−0.09	0.07	0.01	0.24 *	0.05	0.01	0.32 **	0.27 *	−0.01	0.08	-		
14. Mother cohesion	0.06	0.14	0.17	0.36 **	0.16	0.54 ***	0.09	−0.01	−0.08	−0.12	0.03	0.15	−0.17	-	
15. Father consensus	−0.01	0.23 *	0.36 ***	0.41 ***	0.19	0.21	0.08	0.14	−0.09	0.11	−0.24	0.01	−0.08	0.34 **	-
16. Father cohesion	−0.05	0.20	0.11	0.60 ***	−0.19	0.13	−0.03	−0.02	0.12	0.16	0.05	0.15	−0.06	0.49 ***	0.39 ***

Note.

* *p* < 0.05,

** *p* < 0.01,

*** *p* < 0.001.

**Table 4.** Parental relationship qualities and behaviors at age 13 as predictors of emotional support.

	Emotional Support (26)			
	*β* Entry	*β* Final	95% CI	*R* ^2^
*Step 1: Controls*				0.31
Sex	0.20 **	0.25 ***	0.11, 0.39	
Income	0.18 **	0.11	−0.05, 0.27	
Emotional support (21)	0.43 ***	0.40 ***	0.26, 0.53	
*Step 2: Behaviors*				0.33
Mother self-disclosure	−0.05	−0.04	−0.19, 0.10	
Father-reported cohesion	0.09	0.10	−0.16, 0.36	
Mother-reported cohesion	0.08	0.08	−0.19, 0.35	
*Step 3: Qualities*				0.39
Mother valuing	−0.08	−0.08	−0.23, 0.07	
Father-reported consensus	0.21 *	0.21 *	0.01, 0.42	
Mother-reported consensus	−0.16	−0.16	−0.39, 0.07	

Note.

* *p* < 0.05,

** *p* < 0.01,

*** *p* < 0.001.

**Table 5.** Parental relationship qualities and behaviors at age 13 as predictors of acceptance.

	Acceptance (26)			
	*β* Entry	*β* Final	95% CI	*R* ^2^
*Step 1: Controls*				0.21
Sex	0.01	−0.02	−0.17, 0.13	
Income	0.18 *	0.09	−0.08 0.26	
Acceptance (21)	0.41 ***	0.40 ***	0.25, 0.54	
*Step 2: Behaviors*				0.21
Mother self-disclosure	0.07	0.11	−0.05, 0.27	
Father-reported cohesion	0.02	−0.05	−0.34, 0.23	
Mother-reported cohesion	−0.05	−0.06	−0.34, 0.21	
*Step 3: Qualities*				0.24
Mother valuing	−0.09	−0.09	−0.25, 0.08	
Father-reported consensus	0.03	0.03	−0.20, 0.26	
Mother-reported consensus	0.27 *	0.27 *	0.02, 0.51	

Note.

* *p* < 0.05,

*** *p* < 0.001.

**Table 6.** Parental relationship qualities and behaviors at age 18 as predictors of emotional support.

	Emotional Support (26)			
	*β* Entry	*β* Final	95% CI	*R* ^2^
*Step 1: Controls*				0.31
Sex	0.20 **	0.19 *	0.03, 0.35	
Income	0.18 **	0.14	−0.08, 0.36	
Emotional support (21)	0.43 ***	0.49 ***	0.32, 0.65	
*Step 2: Qualities*				0.34
Mother valuing	0.04	0.06	−0.15, 0.28	
Father valuing	−0.16	−0.18	−0.45, 0.09	
Father-reported consensus	0.05	−0.03	−0.29, 0.24	
Mother-reported consensus	0.08	0.08	−0.13, 0.28	
*Step 3: Behaviors*				0.40
Mother self-disclosure	−0.08	−0.08	−0.32, 0.15	
Father self-disclosure	−0.03	−0.03	−0.30, 0.24	
Father-reported cohesion	0.30 *	0.30 *	0.03, 0.58	
Mother-reported cohesion	0.13	0.13	−0.31, 0.20	

Note.

* *p* < 0.05,

** *p* < 0.01,

*** *p* < 0.001.

**Table 7.** Parental relationship qualities and behaviors at age 18 as predictors of instrumental support.

	Instrumental Support (26)			
	*β* Entry	*β* Final	95% CI	*R* ^2^
*Step 1: Controls*				0.24
Sex	0.14	0.14	−0.02, 0.30	
Income	0.17 *	0.00	−0.22, 0.23	
Instrumental support (21)	0.39 ***	0.41 ***	0.26, 0.56	
*Step 2: Qualities*				0.25
Mother valuing	0.06	0.10	−0.12, 0.32	
Father valuing	0.05	0.08	−0.20, 0.37	
Father-reported consensus	0.04	−0.07	−0.35, 0.21	
Mother-reported consensus	0.07	0.05	−0.16, 0.27	
*Step 3: Behaviors*				0.38
Mother self-disclosure	−0.11	−0.11	−0.36, 0.14	
Father self-disclosure	−0.27	−0.27	−0.56, 0.03	
Father-reported cohesion	0.33 *	0.33*	0.02, 0.63	
Mother-reported cohesion	−0.04	−0.04	−0.31, 0.24	

Note.

* *p* < 0.05,

*** *p* < 0.001.

**Table 8.** Parental relationship qualities and behaviors at age 18 as predictors of acceptance.

	Acceptance (26)			
	*β* Entry	*β* Final	95% CI	*R* ^2^
*Step 1: Controls*				0.21
Sex	0.01	0.09	−0.07, 0.26	
Income	0.18 *	0.26 *	0.04, 0.48	
Acceptance (21)	0.41 ***	0.41 ***	0.26, 0.57	
*Step 2: Qualities*				0.23
Mother valuing	0.09	−0.03	−0.26, 0.19	
Father valuing	−0.12	−0.12	−0.42, 0.18	
Father-reported consensus	0.09	0.91	−0.28, 0.30	
Mother-reported consensus	0.02	−0.04	−0.25, 0.17	
*Step 3: Behaviors*				0.29
Mother self-disclosure	0.30 *	0.30 *	0.05, 0.56	
Father self-disclosure	−0.07	−0.07	−0.37, 0.24	
Father-reported cohesion	−0.04	−0.04	−0.36, 0.28	
Mother-reported cohesion	−0.01	−0.01	−0.28, 0.26	

Note.

* *p* < 0.05,

*** *p* < 0.001.

**Table 9.** Parental relationship qualities and behaviors at age 18 as predictors of denial.

	Denial (26)			
	*β* Entry	*β* Final	95% CI	*R* ^2^
*Step 1*				0.10
Sex	0.04	0.01	−0.16, 0.18	
Income	0.03	−0.05	−0.29, 0.19	
Denial (21)	0.32 ***	0.32 ***	0.15, 0.50	
*Step 2: Qualities*				0.22
Mother valuing	−0.19	−0.05	−0.28, 0.18	
Father valuing	0.30 *	0.23	−0.02, 0.47	
Father-reported consensus	0.11	0.29 *	0.02, 0.56	
Mother-reported consensus	0.08	0.13	−0.08, 0.35	
*Step 3: Behaviors*				0.35
Mother self-disclosure	−0.30 *	−0.30 *	−0.54, −0.06	
Father self-disclosure	0.36 **	0.36 **	0.12, 0.61	
Father-reported cohesion	−0.10	−0.10	−0.40, 0.20	
Mother-reported cohesion	0.02	0.02	−0.24, 0.28	

Note.

* *p* < 0.05,

** *p* < 0.01,

*** *p* < 0.001.

## Data Availability

Dataset available upon request from the authors.
